# VESPRO: An Individual Patient Data Prospective Meta-Analysis of Selective Internal Radiation Therapy Versus Sorafenib for Advanced, Locally Advanced, or Recurrent Hepatocellular Carcinoma of the SARAH and SIRveNIB Trials

**DOI:** 10.2196/resprot.7016

**Published:** 2017-02-15

**Authors:** Val Gebski, Emma Gibbs, Mihir Gandhi, Gilles Chatellier, Aurelia Dinut, Helena Pereira, Pierce KH Chow, Valérie Vilgrain

**Affiliations:** ^1^ NHMRC Clinical Trials Centre University Sydney Camperdown Australia; ^2^ NHMRC Clinical Trials Centre University of Sydney Camperdown Australia; ^3^ Biostatistics Singapore Clinical Research Institute Singapore Singapore; ^4^ Clinical Research Unit, Assistance Publique Hôpitaux de Paris Paris France; ^5^ Division of Surgical Oncology National Cancer Centre Singapore Singapore Singapore; ^6^ Department of Radiology, Assistance Publique Hôpitaux de Paris Paris France

**Keywords:** advanced hepatocellular carcinoma, individual patient data prospective meta-analysis, sorafenib, selective internal radiation therapy, noninferiority, percentage of active control retained

## Abstract

**Background:**

Untreated advanced hepatocellular carcinoma (HCC) has an overall poor prognosis. Currently there are 2 ongoing prospective randomized controlled trials that are evaluating the efficacy and safety of sorafenib and selective internal radiation therapy (SIRT) with yttrium-90 resin microspheres in patients with advanced HCC. The SorAfenib versus Radioembolisation in Advanced Hepatocellular carcinoma (SARAH; 459 patients) trial is being performed in Europe and the SIRt VErsus SorafeNIB (SIRveNIB; 360 patients) trial in the Asia Pacific region. Prospectively combining the results, these trials will not only allow for increased precision to estimate efficacy (in terms of survival), but will also provide increased statistical power for subgroup analyses.

**Objective:**

To ensure the prospectivity and transparency of the meta-analysis.

**Methods:**

The sirVEnib and SARAH merge PROject (VESPRO) is an individual, patient-data prospective meta-analysis of the SIRveNIB and SARAH randomized trials. The VESPRO protocol includes prespecified hypotheses, inclusion criteria, and outcome measures. The primary outcome measure is overall survival and secondary outcomes include tumor response rate, progression-free survival, progression in the liver as first event, and disease control in the liver. Pooling of toxicity results will allow for robust safety profiles to be established for both therapies, and provides increased statistical power to investigate treatment effects in key subgroups. Analyses will be performed in the intent-to-treat population stratified by trial.

**Results:**

Both studies are expected to demonstrate a survival benefit for SIRT together with a better toxicity profile compared with sorafenib. It is also anticipated that liver progression as the first event would be longer in the intervention compared with the control.

**Conclusions:**

As the results of the 2 trials are not yet known, the methodological strength is enhanced, as biases inherent in conventional meta-analyses are avoided. This has the effect of providing this meta-analysis with the advantages of a single, large,randomized study of 819 patients. It is anticipated that the SARAH and SIRveNIB trial results will be published separately and together with the combined meta-analysis results from VESPRO. The combined dataset will allow the effect of the interventions to be explored with improved reliability/precision with respect to prespecified patient and intervention-level characteristics.

**Trial Registration:**

Australian New Zealand Trials Registry: ACTRN12617000030370.

## Introduction

Hepatocellular carcinoma (HCC) is the most common type of malignant primary liver tumor, accounting for 80% to 90% of all liver cancers, and most frequently develops in patients with chronic liver disease [1]. HCC is the second leading cause of cancer-related death worldwide and incidence and mortality rates are expected to increase in the coming decades [2,3]. At the time of presentation, the clinical presentation and tumor characteristics of HCC vary considerably; while approximately 40% of HCC patients present with advanced tumors with a high tumor burden or with decompensated liver disease, some patients present with small tumors and compensated chronic liver disease. Thus, the management of HCC is complex, and must take into consideration both patient and tumor characteristics as well as the severity of underlying chronic liver disease.

Curative treatment (by surgical resection, liver transplantation, or radiofrequency ablation) is feasible in very early or early stage HCC, but most patients with intermediate or advanced HCC receive palliative treatment. Advanced HCC is defined as stage C of the Barcelona Clinic Liver Cancer (BCLC) staging system, Eastern Cooperative Oncology Group (ECOG) performance status 1 to 2, portal invasion or extrahepatic spread, and Child-Pugh A-B. The prognosis is poor for patients with untreated advanced HCC, but survival varies depending on the Child‐Pugh score [4-6]. Sorafenib is the only systemic therapy shown to confer survival advantages compared with placebo in patients with advanced unresectable HCC. Two phase III randomized controlled trials (RCTs; the Sorafenib Hepatocellular Carcinoma Assessment Randomized Protocol [SHARP] study and the Asia-Pacific trial) showed significant increases in median overall survival (OS) in patients treated with sorafenib, compared with placebo [7,8]. However, median OS was different in the sorafenib-treated patients from Western countries (SHARP study) and patients from the Asia Pacific region (10.7 months and 6.5 months, respectively). As a result of these data, sorafenib is currently recommended as first-line treatment for advanced HCC [9]. Sorafenib was associated with an overall adverse event incidence of 80%, the most frequent being diarrhea, asthenia, hand-foot reaction, and erythema or desquamation leading, on average, to dose reduction or treatment interruptions in 26% to 44% of patients [7].

Radioembolization (also called selective internal radiation therapy, or SIRT) with yttrium-90 (Y-90) resin microspheres delivered into the hepatic arteries via transfemoral catheterization, is an alternative treatment for advanced unresectable HCC. Several retrospective cohort trials have suggested that SIRT with Y-90 resin microspheres offers similar OS in patients with BCLC stage B or C diseases compared with sorafenib, but with fewer adverse events and better quality of life [10-12]. A recent Cochrane review concluded that there is insufficient evidence to assess the beneficial and harmful effects of Y-90 SIRT for people with unresectable HCC [13]. The authors state that “Further randomised clinical trials are mandatory to better assess the potential beneficial and harmful outcomes of Y-90 microsphere transarterial radioembolisation … for people with unresectable hepatocellular carcinoma” [1].

Several RCTs comparing SIRT with Y-90 resin microspheres with sorafenib for the treatment of HCC are currently underway. The SorAfenib versus Radioembolisation in Advanced Hepatocellular carcinoma (SARAH; [ClinicalTrials.gov identifier NCT01482442]) [14] and SIRt VErsus SorafeNIB (SIRveNIB; [ClinicalTrials.gov identifier NCT01135056]) trials [15] are randomized, open-label phase III studies making head to head comparisons of SIRT and standard of care (ie, sorafenib) in patients with locally advanced HCC. SARAH was performed in France and follow-up is completed, and SIRveNIB is ongoing in countries in the Asia Pacific region. These 2 investigator-based RCTs enrolled patients with advanced or intermediate HCC that did not respond to transarterial chemoembolization with OS as the primary endpoint. Recruitment in these studies is now completed; the survival results by a randomized treatment group and other measures have not yet been reported.

A prospective meta-analysis on individual patient-level data from the SARAH and SIRveNIB studies would increase the power of the studies to assess the treatment effects in this population with advanced HCC and also in key subgroups. This will be useful as treatment effects may differ between patients with HCC in Western and Asian populations. Individual patient data overviews provide more information than conventional meta-analyses. They allow more a detailed investigation and a common statistical analysis plan with an agreement on a standardized methodological approach to the examination.

This study, sirVEnib and Sarah merge PROject (VESPRO), is an individual patient data prospective meta-analysis (IPD-PMA) of the OS survival data of the SARAH and SIRveNIB studies. The primary aim of this meta-analysis is to improve the strength of the evidence on the benefit or potential noninferiority of SIRT compared with sorafenib with respect to OS.

## Methods

### Included Studies

The core trials that make up VESPRO are conducted in accordance with the Declaration of Helsinki and current Good Clinical Practice guidelines, and all participating centers will have obtained the relevant ethics committee approval before patient enrollment.

The primary endpoint of VESPRO is to compare the efficacy of a single SIRT procedure with daily sorafenib, assessed by OS in patients with advanced HCC. Secondary endpoints are to compare the following: cumulative incidence of progression in the liver; progression-free survival (PFS); tumor response rate; disease control rate; and safety and tolerability measured by the incidence of serious adverse events (SAEs).

### Eligible Patients

All patients from the SARAH and SIRveNIB trials will be included in this IPD-PMA. Patients had to satisfy the inclusion and exclusion criteria for the respective trials. These are summarized in Textbox 1 and [14,15].

**Inclusion criteria for respective trials.**

SARAH trial and SIRveNIB trial

- Written informed consent provided
- Aged ≥18 years of age
- Eastern Cooperative Oncology Group (ECOG) performance status 0-1
- Liver cirrhosis Child-Pugh A-B (up to 7 points)
- Adequate hematological function
- Adequate renal function
- Adequate hepatic function

SARAH trial

Histologically or cytologically confirmed diagnosis, or American Association for the Study of Liver Disease criteria for the diagnosis of hepatocellular carcinoma (HCC) and at least one measurable lesion on a computed tomography (CT) scan according to response evaluation criteria in solid tumors (RECIST) criteria

- Patients not eligible for surgical resection, liver transplantation, or radiofrequency ablation who have advanced HCC according to the Barcelona criteria (stage C), with or without portal invasion, or patients with recurrent HCC (new lesion in a different place) after surgical or locoregional treatment who are not eligible for any other treatment or patients in whom chemoembolization has failed after 2 rounds. Treatment failure is defined as the absence of objective response in the treated nodule after 2 rounds (objective response according to the modified RECIST criteria and/or European Association for the Study of the Liver [EASL] criteria)
- Affiliated to a social security scheme or beneficiary

SIRveNIB trial

Unequivocal diagnosis of locally advanced HCC without extrahepatic metastases

- Patients with HCC that is not amenable to surgical resection, immediate liver transplantation, or that could be treated with local ablative techniques (eg, radiofrequency ablation)
- Locally advanced HCC as defined by Barcelona Clinic Liver Cancer (BCLC; B) intermediate stage or BCLC (C) advanced stage.
- At least one lesion that can be accurately measured in at least one dimension (longest diameter to be recorded) as ≥10 mm with spiral CT scan or magnetic resonance imaging (MRI)
- Life expectancy of at least 3 months without active treatment

**Exclusion criteria for respective trials.**

SARAH and SIRveNIB trials

- Advanced liver disease with a Child-Pugh score >B7 or active digestive hemorrhage or encephalopathy or refractory ascites
- Extrahepatic metastases except nonspecific pulmonary tumors <1 cm and abdominal lymph node tumors <2 cm
- Patient unable or unwilling to provide informed consent or comply with the treatment and follow-up required by the trial
- Previously treated advanced hepatocellular carcinoma (excluding chemoembolization)
- Contraindication to hepatic artery catheterisation
- Allergy to trial medications or contrast agents
- Pregnant or breastfeeding women

SARAH trial

- Other primary tumor except for basal-cell carcinomas or superficial bladder cancers
- Unable to take oral medication

SIRveNIB trial

- Intractable ascites, or other clinical signs of liver failure
- Complete thrombosis of the main portal vein
- Other concurrent malignancy, except for adequately treated basal cell or squamous cell skin cancer, in situ cervical cancer, or other cancer for which the patient has been disease free for ≥5 years
- Uncontrolled intercurrent illness
- Currently enrolled in another investigational therapeutic drug or device study
- Men unwilling to use effective contraception during the course of the trial

Adequate hematological function was defined in SARAH as hemoglobin ≥9 g/100 mL, neutrophils ≥1500/mm^3^, platelets ≥50,000/mm^3^, and international normalized ratio () ≤1.5; and defined in SIRveNIB as hemoglobin >9.5 g/dL, leukocytes ≥2500/mm^3^, platelets ≥80,000/mm^3^, and INR ≤2.0. Adequate renal function was defined in SARAH as creatinine <150 µmol/L, and defined in SIRveNIB as albumin ≥2.5 g/dL and creatinine ≤2.0 mg/dL. Adequate hepatic function was defined in SARAH as bilirubin ≤50 µmol/L, aspartate transaminase (AST) or alanine aminotransferase (ALT) ≤5 × upper limit of normal (ULN), and defined in SIRveNIB as bilirubin <2 mg/dL; alkaline phosphatase (ALP), AST, or ALT ≤5 × ULN.

### Study Design

**Figure 1:**
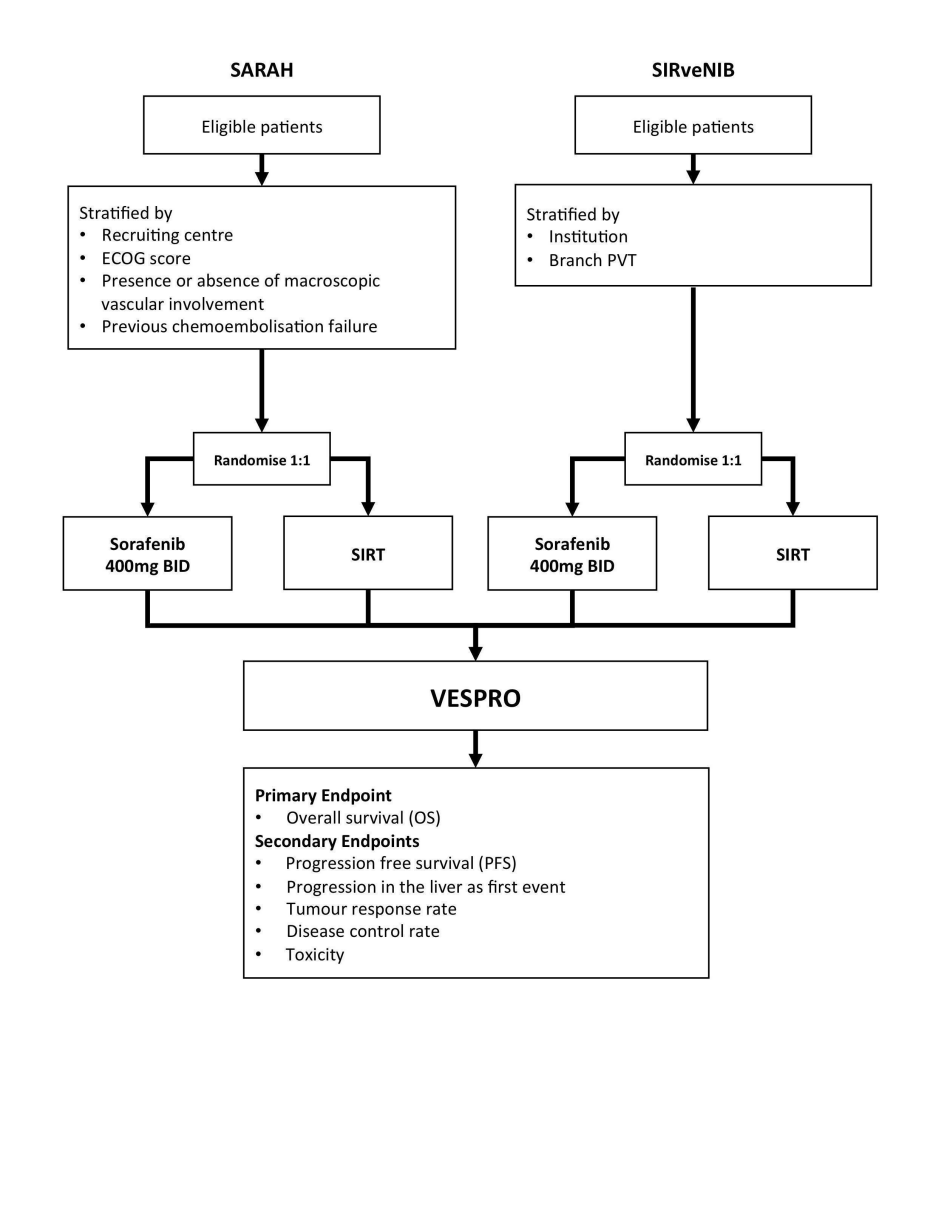
Overview of the VESPO trial design.

VESPRO is an IPD-PMA of the results of the SARAH and SIRveNIB trials (Figure 1). As these trial results are not yet published, this protocol and the corresponding statistical analysis plan were prepared blinded to any trial results, with the aim of documenting methodology and outcomes prior to knowledge of any outcome results from the individual trials.

#### Trials Included in the Meta-Analysis

The SARAH and SIRveNIB trials are randomized open-label trials comparing OS in advanced HCC patients who received SIRT using Y-90 resin microspheres or standard of care (sorafenib). The designs, objectives, and patient recruitment into these trials are summarized in Table 1 [14,15].

**Table 1:** Characteristics of studies included in VESPRO.

Characteristics		SARAH	SIRveNIB
Trial design		Multicenter, open-label, randomized controlled phase III trial comparing SIRT using Y-90 resin microspheres with sorafenib 800 mg/day	Multicenter, open-label, randomized controlled phase III trial comparing SIRT using Y-90 resin microspheres with sorafenib 800 mg/day
Primary objective		To compare the efficacy of Y-90 SIRT with that of sorafenib in the treatment of advanced HCC	To compare the efficacy of Y-90 SIRT with that of sorafenib in the treatment of advanced HCC
Secondary objectives		To compare: Progression free survival (PFS) according to response evaluation criteria in solid tumors (RECIST) and European Association for the Study of the Liver (EASL) at 6 months; tolerability and safety of Y-90 SIRT with those of oral sorafenib; quality of life in the 2 treatment groups; costs in the 2 treatment groups and calculate a cost-effectiveness ratio	To compare: PFS in the liver; PFS at any site; tumor response rate; disease control rate; toxicity and safety; health-related quality of life; liver resection rate; liver transplantation rate; time to disease progression
Primary endpoint		Overall survival (OS)	OS
Secondary endpoints		Adverse events reported according to the National Cancer Institute criteria version 3.0; PFS at 6 months according to RECIST and EASL criteria; response rate (complete, partial or stability) measured according to RECIST and EASL criteria; general and liver disease-specific quality of life scores; cost of each strategy comprising 2 parts: (1) the cost of Y-90 SIRT from the hospital’s perspective; (2) the total cost of each strategy	PFS in the liver; PFS at any site; tumor response rate; disease control rate; toxicity and safety; health-related quality of life; liver resection rate; liver transplantation rate; time to disease progression
**Sample size**			
	Planned	400	360
	Accrued	467	360
	Sample size assumptions	4.3-month increase in median survival from 10.7 to 15 months (hazard ratio [HR] 0.71) 80% power, 95% confidence	4.65-month increase in median survival from 9.35 to 14 months (HR 0.67) 90% power, 95% confidence
Required number of events		Time driven and not event driven	266
Accrual time		24 months	36 months
Follow-up time		12 months	24 months
Randomization		1:1 randomization (stratified blocks).	1:1 randomization (stratified blocks)
Stratification factors		Center; Eastern Cooperative Oncology Group (ECOG) score (0 vs 1); presence of macroscopic vascular invasion (obstruction of the portal vein or its branches); previous chemoembolization failure	Center; presence of branch portal vein thrombosis
**Recruiting countries/ regions**		France	Asia Pacific

#### Treatments

Patients were randomized to either receive sorafenib or SIRT with Y-90 resin microsphere on a 1:1 basis.

In the sorafenib arms, patients received oral treatment with sorafenib (400 mg, twice daily), commencing as soon as possible after randomization in SIRveNIB, but at most within 35 days, and in SARAH within 1 week (7 days) of randomization. Treatment was continued until disease progression, with an anticipated duration of at least 3 months. Treatment suspensions or dose reductions were permitted.

Patients randomized to SIRT were required to have a hepatic angiogram and a liver-to-lung shunt preassessment with technetium-99 m (^99m^Tc)-marked human serum albumin to determine their suitability for the SIRT procedure. The activity of SIRT was calculated using the body surface area or partition model method. SIRT was administrated within 35 days after randomization in SIRveNIB and between 2 and 5 weeks after randomization in SARAH, to allow time for the pretreatment assessments.

#### Trial Schedules

Patients in SIRveNIB were assessed monthly for the first 3 months during protocol treatment and then at 3-month intervals until 24 months following randomization or death. In SARAH, patients were followed up with monthly with an assessment of response every 3 months from randomization until disease progression, death, or the end of the trial. The last enrolled patient was followed up with for up to 12 months after the start of treatment, and all other patients followed up until the final visit of the last enrolled patient; expected duration of patient follow-up was between 12 and 51 months. The full treatment trial schedules are shown in Tables 2 and 3.

**Table 2:** SARAH trial assessment schedule.

Visits	Enrollment	D0^a^	D15	M1^b^	M2	M3	M4	M5	M6	M7	M8	M9	End of participation
Identification	X												
Verification of selection criteria	X												
Consent signature	X												
Initial assessment/history	X												
CT^c^scan	X			X		X			X			X	X
CT perfusion	X			X		X			X				
Lab tests	X	X	X	X	X	X	X	X	X	X	X	X	X
Classification	X			X	X	X	X	X	X	X	X	X	X
Clinical examination				X	X	X	X	X	X	X	X	X	X
Quality of life questionnaires	X			X		X			X			X	X
Preparatory angiography		X											
Scintigraphy		X											
SIRT		X											
Start of sorafenib treatment		X											
Retreatment^d^				X	X	X	X	X	X	X	X	X	
Cancer progression monitoring				X	X	X	X	X	X	X	X	X	X
Sorafenib monitoring				X	X	X	X	X	X	X	X	X	X
Concomitant medication				X	X	X	X	X	X	X	X	X	X
Adverse events				X	X	X	X	X	X	X	X	X	X

^a^D, day.

^b^M, month.

^c^CT, computed tomography.

^d^Timing of retreatment depends upon type of retreatment (see text).

**Table 3:** SIRveNIB trial assessment schedule.

Schedule		Screening/ baseline (eligibility) randomization^a^	During protocol therapy					Trial conclusion	Post trial conclusion follow-up
			Week 2^b^	Week 4	Week 8	Week 12	12-weekly thereafter	As appropriate^c^	12 weekly
	Informed consent	X							
	Demographics	X							
	Medical and surgical history	X							
	Concurrent illness	X							
Concomitant medications^g^		X^d^	X^d,e^	X^d^	X^d^	X^d^	X^d^	X^d^	
**Clinical assessment and physical examination**									
	Height (baseline only)	X		X	X	X	X	X	
	Weight	X		X	X	X	X	X	
	Blood pressure	X		X	X	X	X	X	
	Body temperature	X		X	X	X	X	X	
Performance status	Eastern Cooperative Oncology Group	X		X	X	X	X	X	
**Hematology**									
	Leukocytes	X		X	X	X	X	X	
	Platelets	X		X	X	X	X	X	
	Hemoglobin	X		X	X	X	X	X	
	International normalized ratio (INR)	X		X	X	X	X	X	
**Hepatitis serology**									
	Hepatitis Bsag	X^e^							
	Anti-hepatitis C virus immunoglobulin (IgG )	X^e^							
	Hepatitis B core antibody IgG (optional)	X^e^							
Renal function	Creatine	X		X	X	X	X	X	
**Liver function**									
	Aspartate transaminase (AST)/ alanine aminotransferase (ALT)	X		X	X	X	X	X	
	Alkaline phosphatase (ALP)	X		X	X	X	X	X	
	Total bilirubin	X		X	X	X	X	X	
	Albumin	X		X	X	X	X	X	
	Pregnancy test (as appropriate)	X^f^							
Tumor marker	Serum alpha-fetoprotein (AFP)	X^f^				X	X^f^	X^f^	
EuroQol five dimensions questionnaire (EQ-5D) health-related quality of life		X		X^g^	X	X	X^g^	X^g^	X^g^
Computer tomography or magnetic resonance imaging scan: chest/abdomen/pelvis^h,i^		X				X	X		
**SIRT-arm only**									
	Hepatic angiogram	X^e^							
	^99m^Tc-microaggregated albumin (MAA) lung shunt study	X^e^							
Response assessment^i^						X	X	X	
**Sorafenib arm only**									
	Toxicity assessment		X^b^	X	X	X	X	X	
	Dose delay/modification		X^b^	X	X	X	X	X	
Adverse events (AE)/serious adeverse events (SAE)		AE/SAE for the Sorafenib arm will be recorded from the time of signing the informed consent form (ICF) until 30 days after the final dose of Sorafenib, or until commencement of the next alternative therapy, whichever is earlier. AE/SAE for the SIRT arm will be recorded from the time of signing the ICF until 30 days post-SIRT regardless of causality and for a further 5 months thereafter if judged by the investigator to be causally related to SIRT or Sir-Spheres, or until commencement of the next alternative therapy, whichever is earlier. If the AE/SAE is a Sorafenib- or SIRT-related toxicity follow-up will continue until resolution.							
Survival									X

^a^Screening assessments performed within 28 days before signing of informed consent can be used to confirm eligibility

^b^Sorafenib arm only. Sorafenib patients contacted at week 2 to assess treatment related toxicity and interrupt/modify the dose as necessary

^c^Disease progression, death, complete regression, unacceptable toxicity, patient responds to treatment and becomes eligible for surgical resection, liver transplantation or ablative therapy, lost to follow-up, patient’s request for withdrawal

^d^Concomitant medication to be recorded from screening/baseline up to 30 days post study conclusion (or until commencement of the next alternative therapy, whichever is earlier).

^e^Hepatic angiogram and Tc-99m MAA lung shunt study to be performed after randomization and prior to treatment commencement ONLY for SIRT Arm group

^f^Serum AFP to be performed during screening/baseline and every 12 weeks from date of randomization thereafter. Serum AFP does not need to be repeated for study conclusion visit if it has been performed within the last 28 days.

^g^EQ-5D quality of life questionnaires to be filled out at baseline, while on study (ie, week 4, 8, 12, and every 12 weeks thereafter), at study conclusion, and 12 weekly during post study conclusion follow-up. EQ-5D quality of life questionnaire does not need to be repeated for study conclusion if it has been performed within the last 28 days.

^h^The same radiological assessment method must be used throughout the study.

^i^Assessment for tumor response rate to be done every 12 weeks plus at first disease progression. Radiological assessment for tumor response rate to be done every 12 weeks from date of randomization until first evidence of disease progression.

#### Outcome Measures

The primary endpoint of VESPRO is all-cause mortality measured by OS time. Secondary endpoints include: cumulative incidence of progression in the liver; PFS time; tumor response rate; disease control rate; and incidence of grade 3-4 SAEs. The outcomes are defined in Textbox 3.

**Outcome definitions.**

- Overall survival is defined as the time from randomization to death from any cause, with living patients censored on the date of last follow-up.
- Progression-free survival is defined as the time from randomization until disease progression at any site (response evaluation criteria in solid tumors criteria 1.1) [16] or death. Living patients will be censored on the date of last evaluable tumor assessment.
- Progression in the liver as first event is defined from randomization until the first progression in the liver. Patients alive and progression free will be censored on the date of last evaluable tumor assessment.
- Tumor response rate is defined as the number of patients whose best overall response is complete response (CR) or partial response (PR), divided by the total number of patients in the analysis population.
- Disease control rate is defined as the number of patients whose best overall response is PR, CR, or stable disease, divided by the total number of patients in the analysis population.

#### Toxicity Profile

The toxicity profiles of the 2 groups will be described as the frequency of the worst toxicity grade of adverse event (AE) experienced (according to National Cancer Institute Common Terminology Criteria for Adverse Events). AE rates for the pooled data from the studies will be compared between treatment groups, stratified by trial, using the Mantel-Haenszel technique. The principal comparison will be the proportion of grade 3-4 AEs in each group. In an observational series of 325 patients, SIRT for HCC showed grade 3-4 toxicity profile of: 2.5% fatigue; 1.5% abdominal pain; and 1.5% gastrointestinal (GI) ulceration [17]. In the placebo-controlled SHARP study, patients with advanced HCC who received sorafenib demonstrated serious adverse event/grade 3-4 toxicity profile comprising: 8% diarrhea; 8% hand-foot reaction; 7% liver dysfunction; 5% ascites; 4% other hepatobiliary; 3% fatigue, dehydration, hemoglobin, and cardiac ischemia/infarction; and 2% abdominal pain, hyperbilirubinemia, and weight loss [7]. Other toxicity profiles to be considered include: infection; fever; GI and non-GI bleeding; renal dysfunction; radiation hepatitis; GI ulceration; pulmonary embolism; rash or desquamation; hyponatremia; hypertension; abdominal pain; alopecia; anorexia; ascites; and nausea/vomiting.

### Sample Size Calculation and Statistical Considerations

#### Sample Size Calculation for Individual Trials

In the SARAH trial, hypothetical median survival times, estimated from OS data reported in previous studies [7,18-22], were 10.7 months and 15.0 months in the sorafenib and SIRT arms, respectively, corresponding to a hazard ratio (HR) of 0.71. Enrollment of 400 patients (200 in each treatment arm) would provide 80% power with 95% confidence to detect this risk reduction, based on an accrual period of 24 months and a minimum follow-up of 12 months. The final sample size was 467 patients (459 actually randomized), which allows for an approximately 8.1% rate of patient noncompliance and dropout. The expected number of events was 153 in the SIRT arm and 179 in the sorafenib arm.

In the SIRveNIB trial, the hypothetical median survival times based on OS data reported in previous clinical trials [23,24] were 9.35 and 14.0 months in the sorafenib and SIRT arms, respectively, corresponding to a HR of 0.67. Enrollment of 360 patients (180 per group) would provide 90% power with 95% confidence to detect this risk reduction with an accrual of 36 months and minimum follow-up of 24 months. This sample size also allows for an up to 20% dropout rate. Factoring in this high dropout rate was a pragmatic decision due to the patient recruitment being in developing countries. The expected number of events was 127 in the SIRT group and 139 for the sorafenib group.

#### Prospective Meta-Analysis and Noninferiority

Regardless of the results of the individual trials (statistical significance, or extent of therapeutic benefit), a prospectively designed pooled analysis may help clarify several findings useful for medical decision-making. Thus, the total number of events for the 2 trials combined will provide increased power or precision for assessing the overall treatment effect, and for performing additional analyses among prespecified subgroups. However, pooled analyses resulting in estimates of benefit, which may be small and/or statistically not significant, will raise challenges as to how the results should be clinically interpreted. In this context, a complementary approach is to define a noninferiority (NI) margin to not be appreciably worse clinically **.**

As it is anticipated that both trials will show a benefit, no specific hypotheses will be tested, and issues of statistical power do not arise. The question of interest is whether the 95% confidence interval (CI; one-sided) crosses the NI margin if the pooled result does not reach statistical significance. By exploiting the prospective nature of determining the NI margin, a scientific underpinning can be provided for subsequent clinical interpretation of the results. This approach is based on the assumption that, beside the specific therapeutic actions of SIRT, other aspects of the SIRT intervention could be advantageous, compared with the standard of care (sorafenib). For example, SIRT with Y-90 resin microspheres is administered in a single procedure, while sorafenib is taken daily until disease progression; consequently a better toxicity profile and lower cost could be anticipated with SIRT. In the absence of superiority over sorafenib, SIRT may still be considered a desirable option if the NI boundary is satisfied.

#### Determining the Noninferiority Margin: Fraction of Active Control Retained

To establish a NI margin, the minimum fraction of retained benefit from the active control (sorafenib) is determined. The International Committee on Harmonization E10 guidance from the Food and Drug Administration (FDA) recommends that NI margins should not exceed the smallest effect size that would be expected if the intervention were compared with placebo [25]. The SHARP study showed a 31% risk reduction for mortality with soranifeb versus placebo (HR: 0.69; 95% CI 0.55-0.87) [2]. Likewise, the Asia-Pacific trial showed a 32% risk reduction for mortality with soranifeb versus placebo (HR: 0.68; 95% CI: 0.50-0.93) [8,26]. The pooled overall HR for mortality for sorafenib over placebo is 0.69 (95% CI: 0.57-0.83); or if placebo is compared with sorafenib, there is a 1.46 (95% CI: 1.21-1.75) increase in mortality risk, which equates to an increase of at least 21% (the lower limit of the CI) with placebo over sorafenib. The FDA recommends that the minimum fraction of active control retained should not be lower than 50% [25]. NI margins, based on a one-sided 95% CI, for different fractions of active control retained are shown in Table 4.

**Table 4:** Fractions of active control retained noninferiority (NI) margins.

Active control retained (from a hazard ratio of 1.21)	NI boundary
50%	1.10
70%	1.06
75%	1.05
80%	1.04

A boundary of 10% is considered to be clinically acceptable for potential relative detriment of SIRT compared with sorafenib. Assuming a pooled median survival for sorafenib of 9.5 months, such a margin would translate to an absolute detriment between the 2 groups of less than 5% at 9.5 months. With a 10% NI margin and a fixed sample size of the pooled cohort of 819 with greater than 495 expected events, a survival benefit with SIRT compared with sorafenib of at least 7% (HR: 0.93) would be needed to satisfy this margin at a median survival with sorafenib of 9.5 months.

### Statistical Analyses

#### Primary Endpoint

Statistical analysis of the primary endpoint will be performed in the intent-to-treat population, keeping patients in their randomization groups. The primary outcome (OS) will be compared between treatment arms using the inverse-variance weighted HR of the individual trials. A sensitivity analysis using a stratified log-rank test and an unadjusted stratified proportional hazards model test (stratified by trial) will also be performed. The comparison will be based on superiority. In the event that the 95% CI for the HR crosses the null, if the one-sided upper 95% CI for this HR does not breach the NI boundary of 1.10, this will be interpreted as supporting evidence that SIRT is not appreciably worse than sorafenib.

#### Planned Subgroup Analyses

Subgroup analyses will be performed according to the following baseline characteristics: age (<65 years, ≥65 years); sex; ECOG performance status (0, 1); tumor size (≤50% of liver, >50% of liver); presence or absence of portal vein thrombosis; BCLC stage (B1 and B2, B3 and B4 and C; using Bolondi Criteria); previous treatment for HCC (yes, no); hepatitis status (B, C, both); unilobal versus bilobal disease; single focal versus multifocal disease; and serum alpha-feto protein level (≤100 vs >100 ng/mL).

#### Additional Analysis

As advanced HCC has a poor prognosis, a landmark analysis [27] will be performed at 2 months post-randomization. This conditional analysis will exclude patients that die within 2 months of randomization as such patients are deemed to have disease so severe that neither treatment would be expected to provide any therapeutic benefit.

As SIRT is a locoregional treatment, treatment effect based on progression in the liver as the first event, will be investigated using a competing risk analysis. In this analysis, death or progression outside the liver as the first event will be considered as a competing risk for liver progression. The Gray method [27] will be used to compare groups with HRs and 95% CI estimated from the proportional hazards approach detailed by Fine and Gray [28].

## Results

Patient follow-up in the SARAH trial was completed in March 2016 and patient follow-up in the SIRveNIB trial is currently ongoing (expected completion September 2017).

It is anticipated that the results of SARAH, SIRveNIB, and VESPRO will be published soon after the results are released. As per the individual study protocols, it is anticipated that each study will demonstrate a survival benefit favoring treatment with SIRT. However, practically the intervention could not be given to some patients who were allocated SIRT due to clinical suitability and the results may not be as strongly in favor of SIRT as anticipated. SIRT is a local therapy directed at the liver and the expectation is an increased time to liver progression as the first event in the SIRT cohort compared with sorafenib. It is expected that SIRT will have a lower toxicity profile than sorafenib, which may help guide clinical choice in the event of either or both studies failing to show a significant survival benefit.

## Discussion

### Advantages of Prospective Pooling

The meaningful and relevant data that can be obtained from any clinical trial is restricted by several factors, including the ability to obtain complete outcome data on all (or most) participants, the accuracy and quality of outcomes measurement, and patients’ adherence to the allocated treatment. The SARAH and SIRveNIB trials are evaluating therapies with different modes of delivery; sorafenib is taken orally, twice daily until disease progression or death, whereas SIRT is administered in a single application. The problems of nonadherence that may be encountered with sorafenib include unplanned interruption or cessation of treatment or dose reduction; whereas with SIRT, some patients initially randomized to receive SIRT will be deemed unsuitable after clinical workup and will subsequently receive other treatment. The ability to prospectively pool results from clinical trials in an IPD-PMA will increase the amount of meaningful data available to address important clinical questions.

In a single trial, the ability to draw relevant conclusions from subgroup analyses is also restricted by the low statistical power of the multiple tests in small patient populations. Therefore, there is a disparity between the aim of identifying heterogeneity in the responses of trial participants to treatments and the ability to achieve this goal. Pooling data will provide larger sample sizes that will attenuate the impact of multiple comparisons and enable the detection of small but potentially clinically important differences. The prospective design of such comparisons will add to the credibility of the interpretation of these differences. Additionally, a prospective pooled analysis will facilitate the recognition of signals of clinical interest that in each of the individual trials could potentially be regarded as spurious, and thus disregarded.

If the individual trials and the pooled analysis do not demonstrate statistical significance on the primary endpoint, the question of how the totality of evidence should be interpreted then becomes an issue. These so-called ‘negative results’ may arise due to: a small true benefit; the patients enrolled having a different risk profile to that anticipated; issues with study conduct (nonadherence, lost to follow-up, etc); or changes in clinical practice during the trials. Faced with a ‘negative result’ clinicians may choose to continue with standard care (sorafenib) or introduce the new intervention (SIRT) without strong clinical evidence.

### Defining Margin of Noninferiority Prospectively to Improve Clinical Interpretation

The originality of the VESPRO study is to go beyond classical meta-analysis goals. It is why we propose to consider all the results of the primary and secondary outcomes, and prospective subgroup analyses of the pooled analysis. On a superiority analysis basis, if equality between the 2 treatments is rejected, then due to the greater power the meta-analysis compared with the individual trials a more precise estimate of treatment effects can be provided. If the null hypothesis cannot be rejected, it will be very interesting to determine the reasonable limit within which treatments will be deemed comparable. This margin, while not formally a component of a NI design, will guide interpretation of the results when there is uncertainty. The casual observation suggests that SIRT has a better safety profile than sorafenib, which may be important if efficacy is similar between treatments

Using this information, pooled toxicity profiles and cost estimates will allow clinicians to make informed decisions as to the most appropriate treatment choice for patients with advanced HCC.

## Abbreviations

AE: adverse event
AFP: serum alpha-fetoprotein
ALP: alkaline phosphatase
ALT: alanine aminotransferase
AST: aspartate transaminase
BCLC: Barcelona Clinic Liver Cancer
CI: confidence interval
CR: complete response
CT: computed tomography
ECOG: Eastern Cooperative Oncology Group
EQ05D: EuroQol five dimensions questionnaire
FDA: Food and Drug Administration
GI: gastrointestinal
HCC: hepatocellular carcinoma
HR: hazard ratio
ICF: informed consent form
IgG: immunoglobulin
INR: international normalized ratio
NI: noninferiority
MMA: microaggregated albumin
MRI: magnetic resonance imaging
OS: overall survival
PR: partial response
PFS: progression-free survival
RCT: randomized control trial
RECIST: response evaluation criteria in solid tumors
SAE: serious adverse event
SARAH: sorafenib versus radioembolization in advanced hepatocellular carcinoma
SIRveNIB: selective internal radiation therapy versus sorafenib in locally advanced
SHARP: sorafenib hepatocellular carcinoma assessment randomized protocol
SIRT: selective internal radiation therapy
ULN: upper limit of normal
yttrium-90: Y-90
